# A Novel Finding of Increased ß-Aminoisobutyric Acid Levels in Classic Homocystinuria With Homocysteine-Lowering Treatment

**DOI:** 10.7759/cureus.36911

**Published:** 2023-03-30

**Authors:** Hussam Alkaissi, Samy I. McFarlane

**Affiliations:** 1 Internal Medicine, Kings County Hospital Center, New York, USA; 2 Internal Medicine, Veterans Affairs Medical Center, New York, USA; 3 Internal Medicine, State University of New York Downstate Medical Center, New York, USA

**Keywords:** hyperhomocysteinemia, inborn error of metabolism, myokine, biaba, beta aminoisobutyric acid, cystathionine beta-synthase, classical homocystinuria

## Abstract

Hyperhomocysteinemia is an independent risk factor for cardiovascular disease. Although commonly seen as a milder elevation of homocysteine levels in adult patients, on rare occasions, the internist may face extremely elevated homocysteine levels (>100 µmol/L). In such rare cases, the search for a monogenic disease is warranted. In this report, we present a patient with classical homocystinuria, where the diagnosis was delayed due to various factors. The patient experienced a constellation of symptoms over an extended period, including visual problems, recurrent thrombosis, and neurodevelopmental delay. Delayed diagnosis of genetic diseases is problematic, as patients may grow from pediatric care to adult internal medicine, where knowledge and exposure to such a rare genetic disorder are limited. A diagnosis was finally confirmed with amino acid profiling, revealing extremely elevated homocysteine levels, which were reduced with sequential treatment modalities, including folate, vitamin B12, vitamin B6, methionine restriction, and betaine. We also present derangements in other amino acids, namely, methionine, taurine, serine, and urea cycle products. With treatment, a progressive increase in body weight is noticed. Furthermore, we present a novel finding of increased levels of ß-aminoisobutyric acid with homocysteine-lowering treatment. ß-aminisobutyric acid is a myokine that potentiates some of the metabolic benefits of exercising muscle such as improved insulin resistance and browning of white adipose tissue.

## Introduction

Homocysteine (Hcy) is a byproduct of methionine metabolism and one of four sulfur-containing amino acids, the other three being methionine, cysteine, and taurine. Although methionine, cysteine, and taurine have essential physiological and biochemical functions, homocysteine is very harmful due to forming protein adducts with its cyclical thioester, homocysteine thiolactone (HTL), in a process called N-homocysteinylation of proteins [1]. Homocysteine has caught interest, as it is one of the cardiovascular risk factors and is also associated with low bone density and neurodegenerative disorders such as Alzheimer's disease. Hyperhomocysteinemia (hHcy) refers to elevated blood levels of homocysteine (and its dimer homocysteine). Several disorders can lead to hHcy, most commonly a milder form that results from multiple factors, such as polymorphism of the enzyme methyltetrahydrofolate reductase (MTHFR), cofactor deficiency (folate, vitamin B6, vitamin B12, and the use of antifolate medication such as methotrexate), metabolic syndrome, hypothyroidism, and chronic kidney disease [2]. Rarely, hHcy can be secondary to a Mendelian inherited, single-gene defect of sulfur-containing amino acid metabolism, a condition known as homocystinuria (HCU) [3]. Monogenic, classic homocystinuria is often accompanied by a more extreme elevation of Hcy in blood compared to the more common multifactorial hHcy. Thus, it is associated with more extreme phenotypes (i.e., vascular disease, thrombophilia, bone disease, and neurological decline). Clinically, homocystinuria affects multiple systems with variable presentations. Neurologically, it can cause intellectual and developmental delay, seizures, and behavioral disorders. Osteoporosis with recurrent fractures, marfanoid features, and ectopia lentis is connective tissue and skeletal manifestations that might be related to abnormal cross-linking of sulfhydryl groups of proteins such as elastin, affected by high levels of homocysteine [4]. Although described in the 1960s, insight into the complex pathophysiology of homocysteine disorders has only recently been unfolded. This paper describes a delayed diagnosis of homocystinuria in a patient with classical involvement of the three major systems, vascular, neurological, and skeletal. We also describe secondary metabolic changes of amino acids from baseline and at several time points with treatment with folate, vitamin B12, pyridoxine (vitamin B6), methionine-restricted diet, and betaine. We also present a novel finding of increased ß-aminoisobutyric acid levels after homocysteine-lowering treatment, a marker of exercising muscle with reported anti-inflammatory and anti-insulin resistance effects [5].

## Case presentation

**Figure 1:**
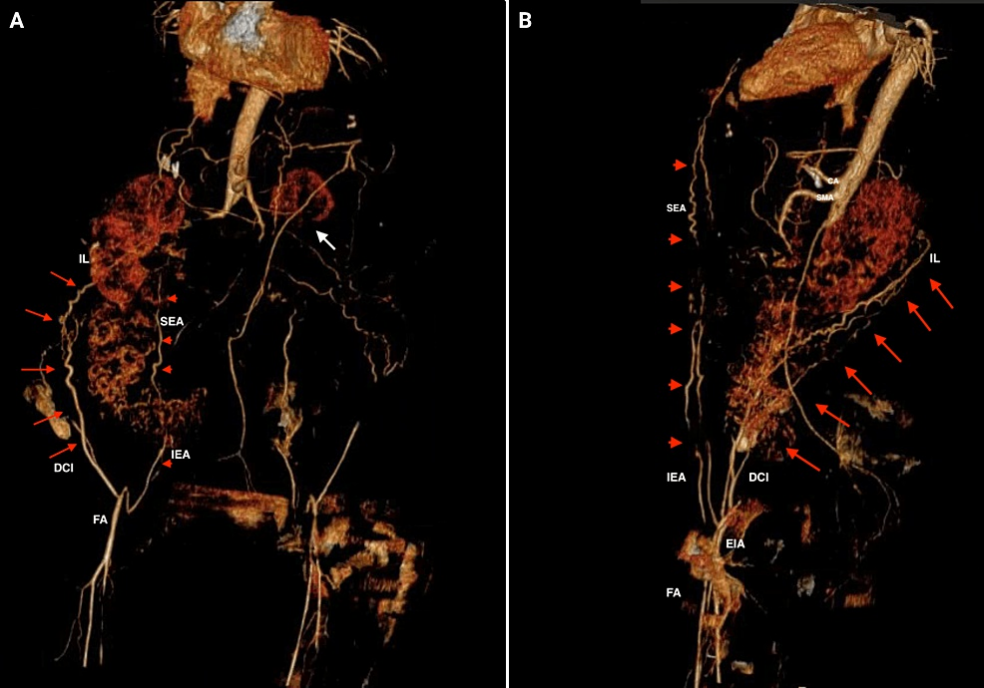
3D reconstruction of computerized tomography angiogram of the aorta and its branches (A) Coronal view showing contrast filling defect below the level of the superior mesenteric artery. Note the partial uptake of contrast in the upper pole of the left kidney due to the extension of the thrombus to part of the left renal artery, leading to infarction of the mid and lower poles (white arrow). (B) Sagittal view. Both views show a resumption of blood flow in the femoral arteries, through two primary anastomoses, first between superior epigastric arteries (SEA) and inferior epigastric arteries (IEA) (red arrowheads), and second anastomosis between iliolumbar (IL) arteries and dorsal circumflex iliac (DCI) arteries (red arrows). CA: celiac artery, DCI: deep circumflex iliac artery, EIA: external iliac artery, FA: femoral artery, SMA: superior mesenteric artery

A 36-year-old woman presented to the emergency department with chest pain for three hours. Initial assessment in the emergency department showed hypertension up to 220/100 mmHg with initial workup included a normal electrocardiogram (ECG) and elevated troponin that was thought to be likely secondary to type 2 myocardial infarction in the setting of a hypertensive emergency. A computerized tomography angiogram of pulmonary arteries was performed, given the history of prior deep venous thrombosis (DVT), but it was unremarkable. CT angiogram showed part of the upper abdominal aorta with incidental finding of filling defect. Therefore, the study was extended to include the aorta and lower extremities, which showed significant aortic thrombosis below the superior mesenteric artery (SMA) level with the involvement of the left renal artery with infarction of the lower and mid poles of the left kidney. The thrombus extended down to the iliac arteries, with a resumption of blood flow in the femoral arteries through two collateral blood supplies (Figure 1). The initial routine biochemical profile was unremarkable (Table 1).

**Table 1:** Routine biochemical profile, including comprehensive metabolic panel, blood count, and troponin I level throughout hospitalization D: day, NA: not available

Variable	D1 on admission	D2 on admission	D3 on admission	D4 on admission	Reference range
Sodium (mmol/L)	137	137	138	135	135-145
Potassium (mmol/L)	3.9	3.5	3.4	3.8	3.5-5.1
Chloride (mmol/L)	103	104	102	105	98-107
Carbon dioxide (mmol/L)	26	24	24	21	21-31
Urea nitrogen (mg/dl)	16	14	16	19	7-25
Creatinine (mg/dl)	0.7	0.8	0.8	0.8	0.6-1.2
Calcium (mg/dl)	8.6	NA	NA	NA	8.2-10
Albumin g/dl)	3.5	3.5	3.6	NA	3.5-5.7
Aspartate aminotransferase (U/L)	NA	35	38	22	10-35
Alanine aminotransferase (U/L)	NA	36	36	26	0-31
Total bilirubin (mg/dL)	NA	0.4	0.6	0.5	0-1.2
Alkaline phosphatase (U/L)	NA	73	82	74	25-125
White-cell count (per µl)	6,960	6,640	7,260	6,570	3,500-10,800
Hemoglobin (g/dl)	13.7	12.9	13.5	12.6	12-16
Platelets count (per µl)	168,000	157,000	163,000	156,000	130,000-400,000
Troponin I (ng/ml)	1.07 0.8 0.32	0.3	<=0.15	NA	<=0.15
Lactic acid (mmol/l)	0.7	NA	NA	NA	0.5-2.2

The patient was admitted and treated for hypertensive emergency; blood pressure was normalized with nitroglycerin infusion over 24 hours, with a resolution of chest pain and troponin elevation.

The patient was born in normal vaginal delivery, uneventful neonatal period with no history of hospitalization. She had delayed developmental milestones. She walked at the age of four years with delayed speech, mostly with incomprehensible words, understood by her family only. Besides the developmental delay, the patient's past medical history consisted of seizure disorder and several thrombotic events (bilateral lower extremities DVT four years prior to presentation). In addition, she has documented bilateral lens dislocations, "ectopia lentis," five years before presentation. She also had a left hip fracture and total hip replacement six years before the presentation. Family history was significant for the sudden death of her father at the age of 48, who had no known medical history. Examination showed arachnodactyly, microcephaly, and a high-arched palate. Examination of the lower extremities showed an absent posterior tibial artery, dorsalis pedis, and popliteal and femoral pulse on palpation. Ultrasound and Doppler studies showed normal flow in lower extremities arteries with smaller spectral waves on Doppler.

**Figure 2:**
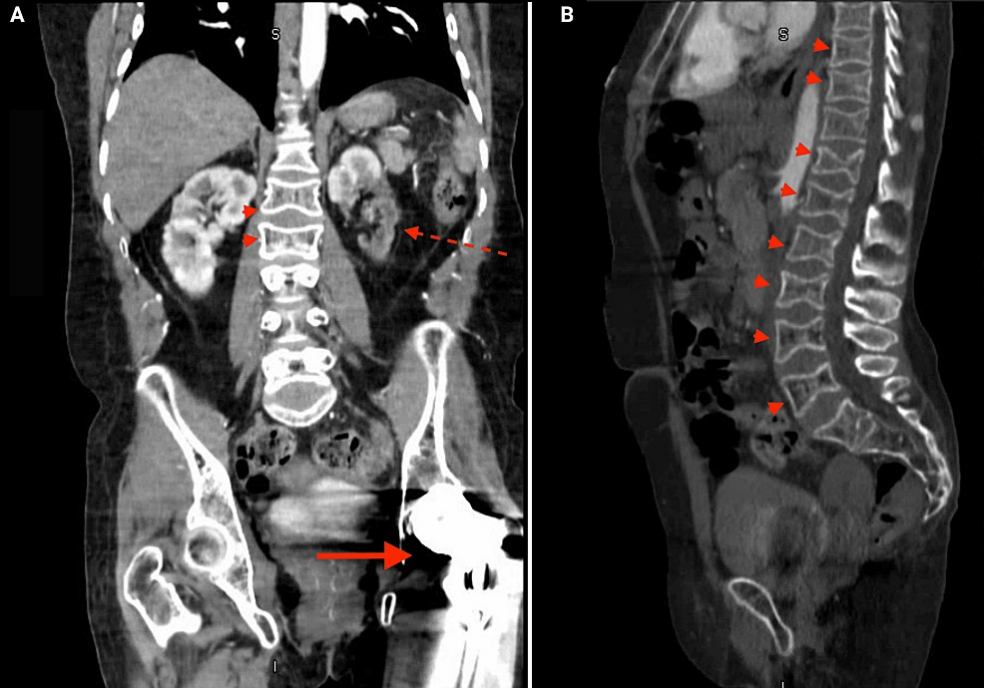
Coronal (A) and sagittal (B) views of computerized tomography of the abdomen and pelvis at the spine level, showing multiple collapsed vertebrae (arrowheads), with osteopenia and artificial hip on the left side (arrow). The infarcted mid and lower poles of the left kidney are also seen (dotted arrow).

**Figure 3:**
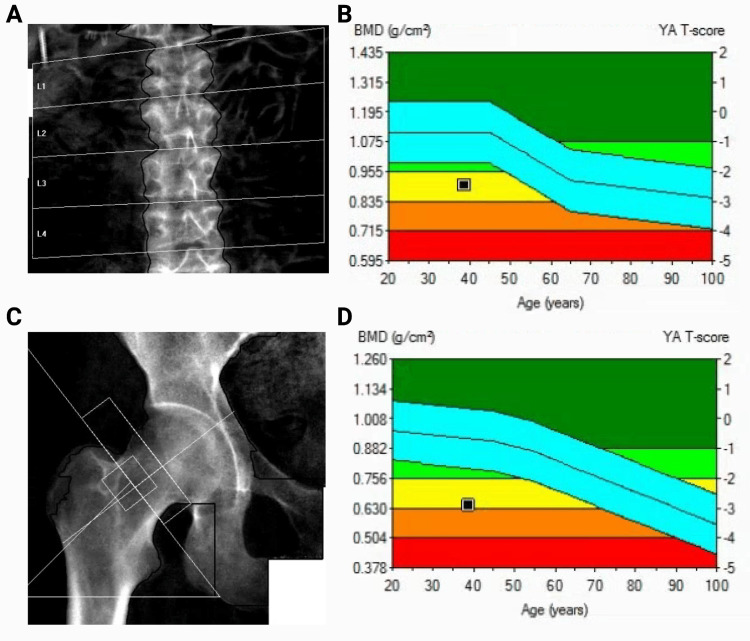
Dual-energy X-ray absorptiometry (DEXA) scan of the lumbar spine (A) with corresponding densitometry (B) and the hip and femoral neck (C) with corresponding densitometry (D) regions showing low bone mineral density as indicated by the mark on densitometry (B and D) lying in the yellow.

Imaging showed diffuse osteopenia in hand radiographs and several vertebral compression fractures and osteopenia (Figure 2, CT). Dual-energy X-ray absorptiometry (DEXA) scan showed low bone mineral density (BMD) (Figure 3), with lumbar spine Z-scores of -1.7, femoral neck Z score of -2.3, and hip Z-score of -2.2 (Table 2). Electroencephalogram (EEG) showed a slow posterior dominant rhythm, with diffuse background slowing and occasional bursts of polymorphic delta waves with higher amplitude over the right hemisphere with independent bitemporal polymorphic slowing. These EEG findings suggest diffuse cerebral dysfunction with independent bitemporal dysfunction.

**Table 2:** Bone density measured in DEXA and expressed as g/cm2 of lumbar vertebrae (L1-L4) and hip region. T-scores and Z-scores of corresponding densities are shown. BMD: bone mineral density, DEXA: dual-energy X-ray absorptiometry

Region	BMD (g/cm^2^)	T-score	Z-score
L1	0.803	-2.8	-2.1
L2	0.908	-2.5	-1.8
L3	0.960	-2.1	-1.4
L4	0.899	-2.5	-1.8
L1-L4	0.897	-2.4	-1.7
Femoral neck	0.612	-3.1	-2.3
Total hip	0.641	-2.9	-2.1

Thrombophilia workup was unremarkable, including levels and activity of protein C, protein S, factor V Leiden, antithrombin, factor II 20210A, and antibodies to antiphospholipid syndrome (lupus anticoagulant, anticardiolipin antibodies, anti-ß2-glycoprotein antibodies, IgG, IgM, and IgA). After obtaining records from different hospitals that included information about previous thrombotic events, and the ectopia lentis, we measured homocysteine levels, and initial levels were extremely elevated at >500 µmol/L (reference range <10 µmol/L), establishing the diagnosis of homocystinuria (HCU). In addition, fasting homocysteine levels were elevated at 106 umol/l (reference < 1 umol/L) as well as fasting methionine levels elevation at 170 umol/l (reference 16-34 umol/l) was documented (Appendices). Family members were screened with homocysteine and methionine measurements (sister and mother), and both had normal levels.

**Figure 4:**
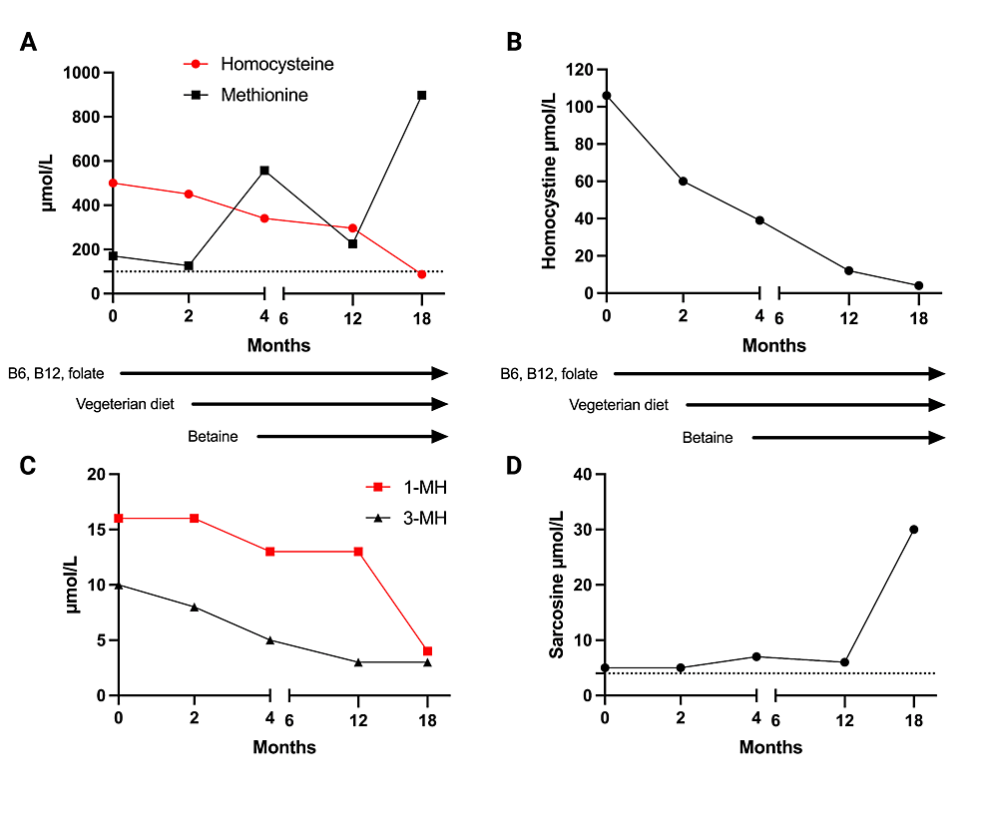
(A) Initial homocysteine levels (red) >500 µmol/L, with subsequent reduction to levels below 100 µmol/L (dotted line), a level desired to prevent further thrombotic events. Methionine levels (black) increase with treatments that boost homocysteine remethylation. (B) Homocysteine levels (homocysteine dimers) were reduced with several lines of treatment. (C) Levels of 1-methylhistidine (red) and 3-methylhistidine (black) were reduced after introducing a vegetarian, methionine-restriction diet. (D) Sarcosine levels increase a marker of betaine treatment. 1-MH: 1-methylhistidine, 3-MH: 3-methylhistidine Figure created with GraphPad Prism 9

After discharge, she was started on vitamin B6 (pyridoxine) 10 mg/kg/day (total of 500 mg daily) to assess responsiveness to vitamin B6, with serial measurements of homocysteine (Hcy). She was also started on folate 1 mg daily and vitamin B12 1000 µg daily, given an initial elevation of methylmalonic acid. Hcy levels reduced to 280s µmol/L but still above the desired target of ≤100 µmol/L, indicating partial responsiveness to B6. The patient then was switched to a vegan, low-methionine diet (excluding meat, dairy products, and eggs) with amino acid formula deficient in methionine. The dietary intervention led to a milder reduction in Hcy and Hcy-Hcy levels (Figures 4A, 4B). With the exclusion of meat products, 1-methylhistidine and 3-methylhistidine levels reduced over time (Figure 4C). The introduction of betaine led to further improvement of homocysteine levels, eventually achieving a desired target of <100 µmol/L, accompanied by an increase in sarcosine, a byproduct of demethylated betaine and a marker of betaine use compliance (Figure 4D). Follow-up over two years showed improved energy level and behavior with sleep-wake cycle regulation. Before treatment, the patient had bouts of agitation and irritability at night, sometimes associated with self-harm, such as head banging against the wall in a repeated manner. Most of these behaviors are resolved with treatment. She could maintain longer conversations with her family, listen to music, and enjoy watching television with a longer attention span. She acquired more words in English than she used to communicate with her family.

**Figure 5:**
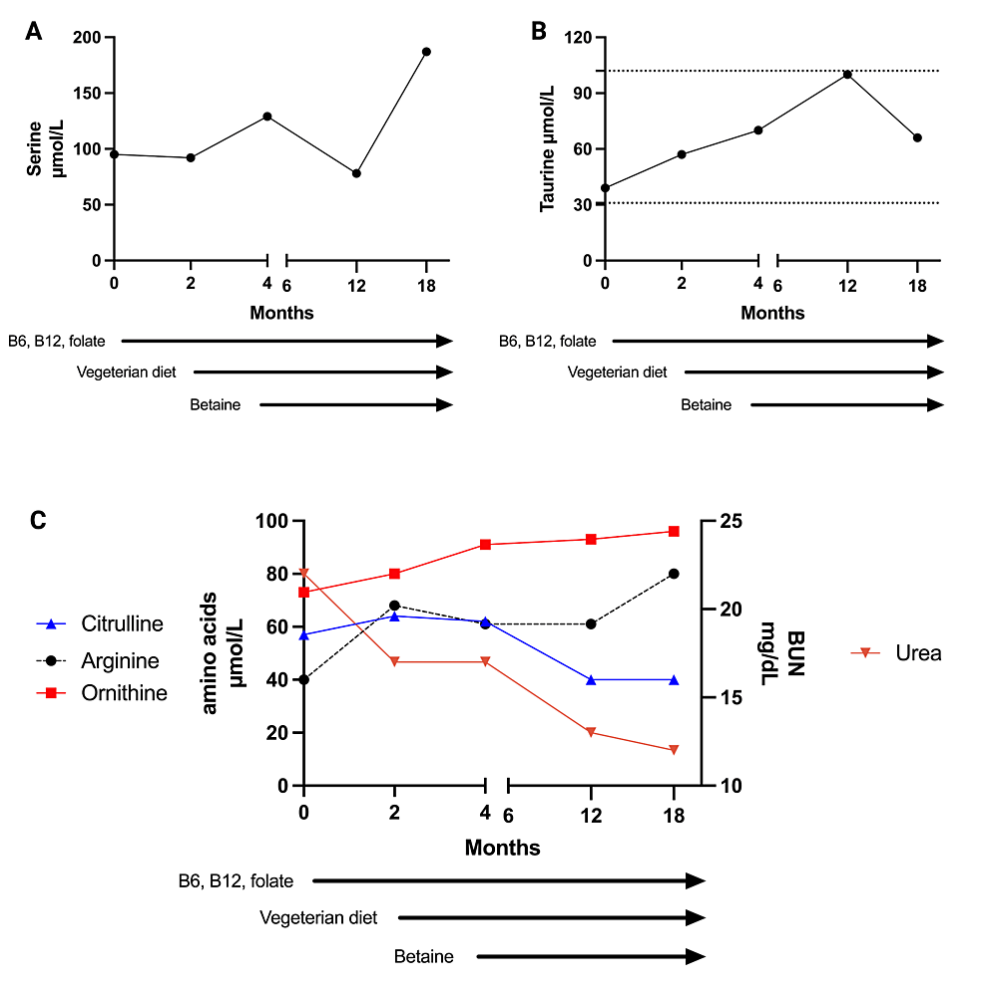
(A) Serine levels increased with treatment. (B) Taurine levels increase after introducing vitamin B6, a product of the transsulfuration pathway. (C) Changes in urea cycle amino acids throughout treatment, namely, arginine, ornithine, citrulline, and blood urea nitrogen. Figure created with GraphPad Prism 9

We followed amino acid levels and showed serial improvement of initially low serine levels increase in taurine and ß-aminoisobutyric acid. In addition, amino acids from the urea cycle were affected during treatment (Figure 5).

**Figure 6:**
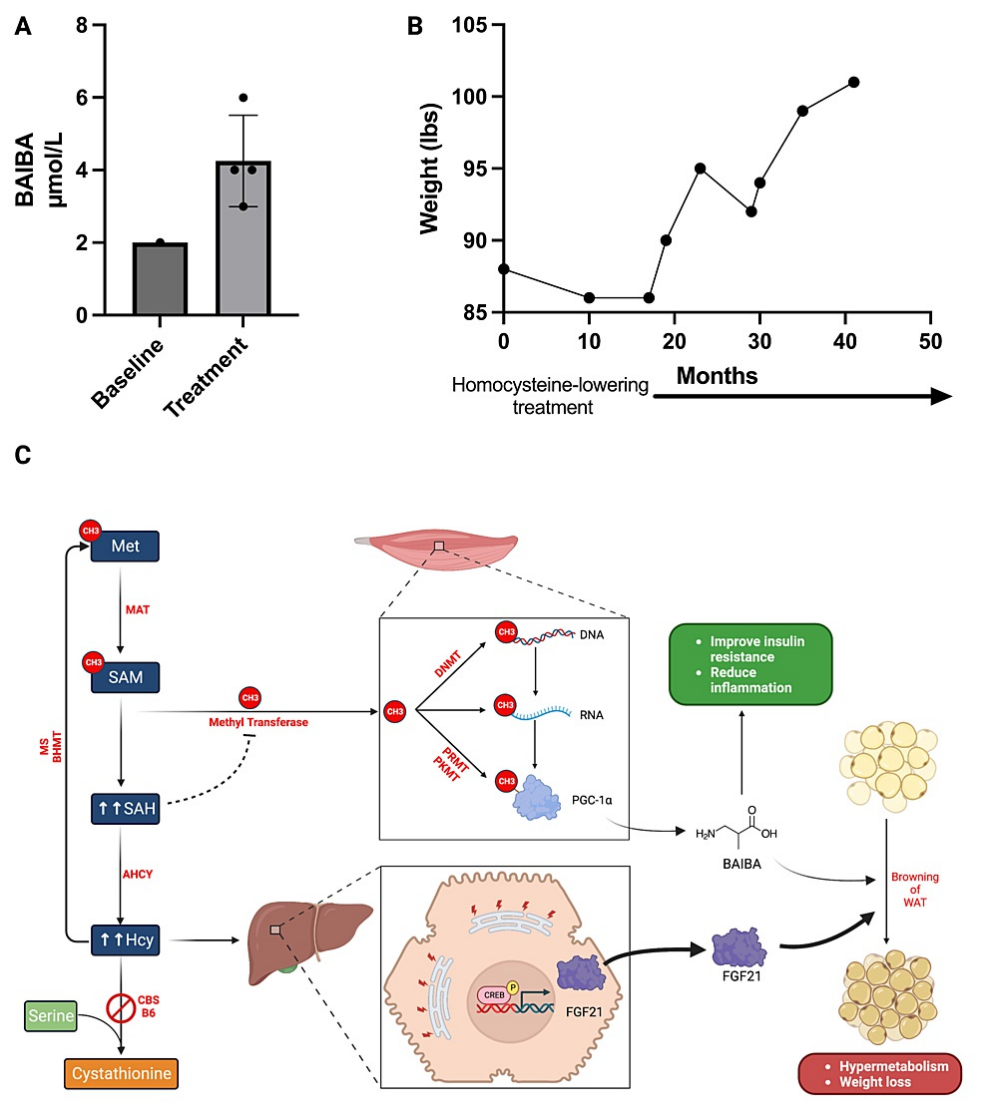
After diagnosis and treatment with homocysteine-lowering agents (vitamin B6, B12, folic acid, methionine restriction, and betaine), a noticeable increase in ß-aminoisobutyric acid (BAIBA) levels (A) and total body weight (B) was seen. (C) Potential mechanisms for increasing BAIBA include improved exercise capacity, reduced clots burden, improved muscle perfusion in the lower extremities, and an increase in PGC-1α methylation, a regulator of BAIBA synthesis. The mechanism for lower body weight in homocystinuria may involve hepatocyte ER-stress-included increase in FGF21 levels, which promotes the browning of white adipose tissue and hyper-metabolism, leading to a phenotype of lipodystrophy and weight loss. AHCY: adenosylhomocysteinase, BAIBA: ß-aminoisobutyric acid, BHMT: betaine-homocysteine methyltransferase, CBS: cystathionine-ß-synthase, CH3: methyl group, CREB: cAMP response element binding protein, DNMT: DNA methyltransferase, FGF21: fibroblast growth factor 21, MAT: methionine adenosyltransferase, MS: methionine synthase, PGC-1α: peroxisome proliferator-activated receptor-gamma coactivator-1α, PKMT: protein lysine methyltransferase, PRMT: protein arginine methyltransferase, WAT: white adipose tissue Figures 6A, 6B created with GraphPad Prism 9; Figure 6C created with BioRender.com

Closer follow-up for two years after diagnosis and lowering of homocysteine levels, levels of the myokine beta-aminoisobutyric acid levels increased (Figure 6A). Total body weight also increased over a period of 20 months, gaining about 13 pounds (Figure 6B).

## Discussion

This report presents a patient with classical homocystinuria, where the diagnosis was delayed due to multiple factors. First, the patient was born in a country where homocystinuria screening was unavailable. Second, the constellation of symptoms was presented over a prolonged time and addressed by different specialists without a unifying diagnosis. These symptoms include visual problems and lens dislocation, recurrent thrombosis, and the life-long neuro-developmental delay attributed to post-infectious encephalitis. This case highlights the problem with delayed diagnosis of genetic disorders where the patient grows from pediatric care to adult care, where knowledge of and expertise with such orphan diseases and rare genetic disorders are generally quite limited.

In this case, the diagnosis was confirmed with amino acid profiling, and we measured 35 amino acids (including non-proteinogenic amino acids) over four-time points during homocysteine-lowering treatment. Following the introduction of a low-methionine diet, there is an anticipated reduction in 1-methylhistidine (1-MH) and 3-methylhistidine (3-MH), which are markers of meat intake (Figure 4C) [6,7].

Interestingly, serine levels were increased with treatment (vitamin B6 and betaine). Serine has multiple roles in homocysteine metabolism. It has two roles in the remethylation pathway and a third in the transsulfuration pathway, all with reducing effects on HCy levels. First, serine is the source of endogenous betaine (through the ethanolamine-choline-betaine pathway). Second, serine can directly methylate tetrahydrofolate (THF) to N5, N10-methylene-THF (the latter is converted to 5-methyl-THF by MTHFR), producing glycine as a byproduct. Both of these pathways result in the remethylation of HCy to methionine. Third, serine has a role in the transsulfuration pathway by condensing with HCy to form cystathionine, catalyzed by the cystathionine-ß-synthase (CBS) enzyme. Therefore, in our patient, serine levels were relatively in the lower normal range before any treatment, indicating a possible pressure on the serine pool to aid in remethylation, as indicated by elevated methionine and sarcosine levels at baseline before any treatment. Then, with B6 and exogenous betaine treatment, the serine level increases. This phenomenon of the serine-homocysteine axis has been described before by Dudman et al. in 1987, where 16 patients with classical HCU (secondary to CBS defect) had mean serine levels of 91 µmol/L (±18 µmol/L) compared to healthy adults with mean serine levels of 121 µmol/L (±25 µmol/L). HCU patients' serine levels increase to normal upon treatment with betaine. The explanation is that the serine pool is indirectly shuttled to remethylate HCy by remethylating THF and de novo synthesis of endogenous betaine. This is also evident in reduced serine levels in healthy individuals by 13% with folate supplementation. Similarly, patients with impaired renal function and elevated HCy levels also have lower serine levels by 17% compared to healthy adults (Figure 5A) [8].

With the normalization of serine, ethanolamine, a main product of serine metabolism, also increased. Ethanolamine is critical, as it is a precursor to choline that is further utilized to synthesize acetylcholine; a major neurotransmitter, betaine, with its role in remethylation, and phosphatidylcholine, a major phospholipid of the cell membrane. That explains the low levels of ethanolamine in neurological disorders such as Alzheimer's disease and major depressive disorder [9,10]. Similarly, lower serine levels are linked to abnormal phospholipid and sphingolipid metabolism, mediated by lower ethanolamine levels, leading to impaired nervous system development and function [11]. However, it is unknown if low ethanolamine is a known association with homocystinuria, given the state of functional serine deficiency, as it is being shuttled to excess homocysteine methylation and whether it has a role in the neurological manifestation of homocystinuria.

Taurine, an amino-sulfonic acid and downstream product of the transsulfuration pathway, was increased in our patient after vitamin B6 supplementation, likely secondary to some residual enzymatic activity in the CBS enzyme. Taurine levels are generally abundant in the central nervous system and may reduce neuronal activities, given its structural similarities to the inhibitory neurotransmitters gamma-aminobutyric acid (GABA) and glycine, yet, its exact function as a neurotransmitter is still debatable [12]. Therefore, some of the neurological improvement in our patient might be secondary to an increase in taurine levels. Furthermore, taurine is also involved in bile acids synthesis, thus reducing cholesterol levels, which may have endothelial and general cardiovascular favorable effects in our patient. In addition, preclinical data indicate that taurine may help increase the expression of hepatic betaine-homocysteine methyltransferase (BHMT), the main remethylation pathway in betaine-treated HCU patients. BHMT expression is lost with time, as well as betaine efficacy; thus, taurine treatment may salvage such cases [13].

We examined products of the urea cycle and found that citrulline levels were initially mildly elevated but normalized with treatment (betaine), though the causality is unclear (Figure 5C). Of note, the mice model of HCU (Cbs -/-) has significantly elevated citrulline (2.2 folds) and ornithine (2.7 folds) as compared to wild-type (WT) mice [14]. A low protein diet might be the cause of such lower levels. Ornithine levels, on the other hand, increased with treatment.

We measured ß-aminoisobutyric acid (BAIBA) levels before and after homocysteine-lowering treatment. We found low levels of BAIBA at baseline that doubled after treatment, as seen on multiple occasions (Figure 6A). BAIBA is a myokine secreted from muscles after exercise (mediated by PGC-1α), leading to stimulation of brown fat through PPARα. BAIBA's benefits include increased insulin sensitivity, reduced hepatic very low-density lipoprotein (VLDL) production, and increased bone density [15].

Our patient had an increase in BAIBA levels after treatment, which can be explained by increased exercise capacity or by improved hyperhomocysteinemia and resolution of an aortic clot that minimized femoral arterial blood flow. BAIBA is produced from the amino acid valine or the nucleic acid thymine, downstream from PGC-1α signaling in the exercising muscle. Evidence suggests PGC-1α methylation is important for its nuclear localization and signaling. This is evident in cases of defective one-carbon metabolism and general state methyl donor deficiency, as seen in deficiency of folate and vitamin B12 leads to altered S-adenosylmethionine (SAM)/S-adenosylhomocysteine (SAH) ratio and reduction of PGC-1α methylation at arginine residues, resulting in a reduction in its function [16,17]. As such, folate and vitamin B12 supplementation improves PGC-1α methylation and target gene expression and may increase BAIBA levels (Figure 6C). Such a defect in PGC-1α methylation may partly be involved in bone disease in homocystinuria, as a hypomethylated PGC-1α has been shown to interact with vitamin D receptor (VDR), affecting bone density [18]. Interestingly, PGC-1α also regulates key enzymes in homocysteine metabolism such as methionine adenosyltransferase (MAT), adenosylhomocysteinase (AHCy), and betaine-homocysteine methyltransferase (BHMT). These effects are mediated by HNF4α [19].

Finally, with treatment and lowering of homocysteine levels, our patient gained around 13 pounds though her body mass index (BMI) was still less than 20 (Figure 6B). Homocystinuria patients tend to have lower body weight in general [20]. Studies on animal models with elevated homocysteine due to BHMT knockout have shown that the weight loss is due to increased fibroblast growth factor 21 (FGF21) levels secondary to homocysteine-induced hepatocyte endoplasmic reticulum (ER) stress. Such an increase in FGF21 promotes the browning of white adipose tissue and hyper-metabolism, leading to a phenotype of lipodystrophy [21]. A cross-sectional study of individuals with inborn errors of metabolism, including homocystinuria, showed an increase in FGF21 levels, consistent with preclinical findings [22].

## Conclusions

In this paper, we discuss the diagnosis and treatment of classic homocystinuria, an inborn error of homocysteine metabolism, with serious cardiovascular, neurological, and skeletal outcomes if the diagnosis is missed. The disease is commonly encountered in pediatric age groups but could be missed, especially if screening for the disease is unavailable. Adult health care providers, including internists, should be aware of the possibility of the diagnosis, especially if constellations of clinical manifestations are present with highly elevated homocysteine levels.

In this report also, we presented comprehensive proteinogenic and non-proteinogenic amino acids profiles at baseline prior to treatment and throughout the treatment course, showing the complexity and interaction of a large system of biochemical pathways, including serine, taurine, and urea cycle products. We also presented a novel finding of increased levels of the advantageous myokine and exercise marker ß-aminoisobutyric acid in a patient with homocystinuria after homocysteine-lowering treatment.

## Appendices

**Table 3:** Amino acid profile, vitamin B12, folate, and methylmalonic acid levels at baseline and after the introduction of vitamin B6, float, and vitamin B12, then exclusion of methionine-rich dietary items (2 months), then the introduction of betaine BUN: blood urea nitrogen, MMA: methylmalonic acid, Ig: immunoglobulin, NA: not available

Variable	(baseline)	2 months)	(4 months)	(12 months)	(18 months)	Reference range
Homocysteine	>500	450	340	296	87	<10.4
Homocystine	106	60	39	12	4	<1
Methionine (µmol/L)	170	126	557	225	898	16-34
Cystathionine (µmol/L)	<1	<1	<1	<1	<1	<1
Taurine (µmol/L)	39	57	70	101	66	31-102
Sarcosine (µmol/L)	5	5	7	6	30	<4
MMA (nmol/L)	547	NA	244	148	125	87-318
Folic acid (ng/mL)	4.6	6.3	NA	NA	16	3-16
Vitamin B12 (pg/mL)	209	513		996	1849	165-1000
Alanine (µmol/L)	298	414	475	293	397	200-482
Proline (OH) (µmol/L)	15	20	19	8	6	4-27
Proline (µmol/L)	172	218	422	175	245	104-383
Threonine (µmol/L)	150	183	150	114	94	67-198
Serine (µmol/L)	95	92	129	78	187	65-138
Ethanolamine (µmol/L)	<4	6	NA	5	<4	5-13
Glycine (µmol/L)	286	416	349	300	295	122-322
Aspargine (µmol/L)	55	61	38	71	66	31-64
Glutamine (µmol/L)	708	694	499	550	578	428-747
Aspartic a (µmol/L)	3	1	1	4	6	1-4
Glutamic acid (µmol/L)	32	36	99	49	60	10-97
Histidine (µmol/L)	81	110	104	102	61	60-109
1-methyl-histidine (µmol/L)	16	<1	13	13	4	<47
ß-alanine (µmol/L)	4	<1	4	4	2	<5
3-methyl-histidine (µmol/L)	10	5	5	3	3	2-9
Lysine (µmol/L)	156	152	174	144	179	199-233
α-aminoadipic acid (µmol/L)	<1	<1	2	2	NA	<2
Arginine (µmol/L)	40	68	61	61	80	43-107
Ornithine (µmol/L)	73	80	91	93	96	27-83
BUN (mg/dL)	22	17	17	13	12	7-25
Citrulline (µmol/L)	57	64	62	40	40	16-51
Valine (µmol/L)	225	176	263	182	197	132-313
Leucine (µmol/L)	93	74	131	132	74	73-182
Isoleucine (µmol/L)	51	44	71	33	36	34-98
Tyrosine (µmol/L)	50	59	87	61	66	38-96
Phenylalanine (µmol/L)	52	68	65	95	67	40-74
Tryptophan (µmol/L)	50	48	69	63	64	40-91
α-amino-N-butyric acid (µmol/L)	26	19	19	16	20	7-32
ß-aminoisobutyric acid (µmol/L)	2	6	4	4	3	<3
γ-aminobutyric acid (µmol/L)	<1	<1	<1	<1	<1	<3
